# Fine-tuning the tRNA anticodon arm for multiple/consecutive incorporations of β-amino acids and analogs

**DOI:** 10.1093/nar/gkae219

**Published:** 2024-04-04

**Authors:** Takayuki Katoh, Hiroaki Suga

**Affiliations:** Department of Chemistry, Graduate School of Science, The University of Tokyo, 7-3-1 Hongo, Bunkyo-ku, Tokyo 113-0033, Japan; Department of Chemistry, Graduate School of Science, The University of Tokyo, 7-3-1 Hongo, Bunkyo-ku, Tokyo 113-0033, Japan

## Abstract

Ribosomal incorporation of β-amino acids into nascent peptides is much less efficient than that of the canonical α-amino acids. To overcome this, we have engineered a tRNA chimera bearing T-stem of tRNA^Glu^ and D-arm of tRNA^Pro1^, referred to as tRNA^Pro1E2^, which efficiently recruits EF-Tu and EF-P. Using tRNA^Pro1E2^ indeed improved β-amino acid incorporation. However, multiple/consecutive incorporations of β-amino acids are still detrimentally poor. Here, we attempted fine-tuning of the anticodon arm of tRNA^Pro1E2^ aiming at further enhancement of β-amino acid incorporation. By screening various mutations introduced into tRNA^Pro1E2^, C31G39/C28G42 mutation showed an approximately 3-fold enhancement of two consecutive incorporation of β-homophenylglycine (βPhg) at CCG codons. The use of this tRNA made it possible for the first time to elongate up to ten consecutive βPhg's. Since the enhancement effect of anticodon arm mutations differs depending on the codon used for β-amino acid incorporation, we optimized anticodon arm sequences for five codons (CCG, CAU, CAG, ACU and UGG). Combination of the five optimal tRNAs for these codons made it possible to introduce five different kinds of β-amino acids and analogs simultaneously into model peptides, including a macrocyclic scaffold. This strategy would enable ribosomal synthesis of libraries of macrocyclic peptides containing multiple β-amino acids.

## Graphical Abstract

**Graphical Abstract:**
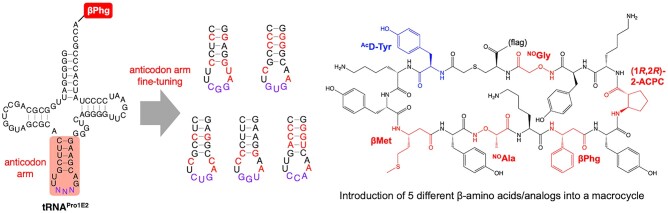

## Introduction

Ribosomal incorporation of various nonproteinogenic amino acids into peptides and proteins has been enabled by development of genetic code manipulation methodologies, such as nonsense codon suppression (1), sense codon reassignment (2) and quadruplet codon suppression (3). l-α-Amino acids with sidechain modifications can be relatively efficiently introduced into nascent peptide chains, whereas incorporation of backbone modified amino acids, such as β-amino acids, is generally much less efficient (4–9). Moreover, multiple and/or consecutive incorporation of diverse β-amino acids at once is by far more difficult compared to incorporation of a single, selected β-amino acid (8). In fact, multiple/consecutive incorporation of β-amino acids had been considered impossible for a long time. On the other hand, it has been known that β-amino acids possess stronger turn/helix inducing abilities than α-amino acids, and thereby peptides comprised of β-amino acids can fold into unique and stable structures, referred to as foldamers (10–12). Such peptides could exhibit enhanced binding affinity and specificity to target molecules (13–15), membrane permeability (16,17), and proteolytic stability (18–21). Thus, β-amino acids are an attractive set of building blocks to develop novel bioactive peptides.

Selection approach by a combination of genetic code reprogramming powered by the flexizyme technology (22) and mRNA display (23,24), referred to as RaPID (Random nonstandard Peptides Integrated Discovery) system (25), has led to the discovery of *de novo* macrocycles containing nonproteinogenic amino acids (25–30). During the course of these studies, however, we have also observed some nonproteinogenic amino acids, such as β-amino acids and d-α-amino acids, are poorly incorporated using well-established suppressor tRNAs (e.g. tRNA^AsnE2^), particularly when their consecutive incorporation occurs (8,31). With this reason, we have been devoting our efforts to improve their incorporation efficiency, thereby allowing us to construct libraries containing such nonproteinogenic amino acids and to discover *de novo* bioactive peptides using the RaPID system.

**Figure 1.**
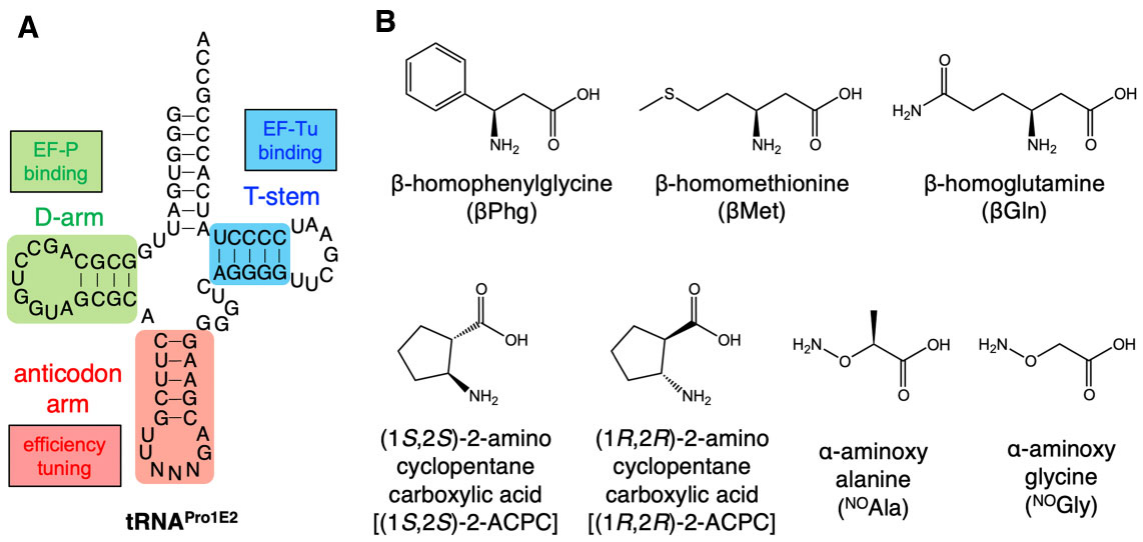
Structures of tRNA and amino acids used in this study. (**A**) Secondary structure of tRNA^Pro1E2^. EF-P and EF-Tu recognize the D-arm and T-stem motifs indicated by green and blue, respectively. Anticodon arm is indicated by red. (**B**) Structures of β-amino acids and β-amino acid analogs used in this study. Three β^3^-amino acids (βPhg, βMet and βGln), two cyclic β^2,3^-amino acids [(1*S*,2*S*)-2-ACPC and (1*R*,2*R*)-2-ACPC] and two α-aminoxy acids (^NO^Ala and ^NO^Gly).

The inefficiency of β-amino acid incorporation could be mainly attributed to the following two reasons: (i) the inefficient accommodation of β-aminoacyl-tRNA onto the ribosomal A site and (ii) the slow peptidyl transfer reaction of β-aminoacyl-tRNAs (32). To overcome these issues, we have previously developed a designer tRNA, referred to as tRNA^Pro1E2^, bearing specific T-stem and D-arm motifs that can be recognized by EF-Tu and EF-P, respectively (Figure 1A) (33,34). β-Aminoacyl-tRNA^Pro1E2^ can be efficiently accommodated onto ribosome by EF-Tu and the peptidyl transfer reaction of them can be accelerated by EF-P. By using the tRNA^Pro1E2^ body sequence, we have demonstrated consecutive incorporation of β-amino acids into peptides (26,29,32). Moreover, tRNA^Pro1E2^ has allowed us to incorporate β-amino acid analogs, such as α-aminoxy acids and α-hydrazino acids, into nascent peptide chain, yielding unique backbone alternative peptides in ribosomal expression ever (35). However, even if tRNA^Pro1E2^ is used, simultaneous introduction of multiple kinds of β-amino acids into nascent peptide is still poorly achieved, and thus it remains a major challenge.

Although the sequences of T-stem and D-arm of tRNA^Pro1E2^ have been optimized for binding to EF-Tu and EF-P, respectively, other regions of this tRNA have not yet been finetuned for β-amino acid incorporation. It has been previously suggested that the sequence of anticodon arm could affect the efficiency of translation; for instance, Yarus *et al.* investigated amber suppressor tRNA variants bearing anticodon stem/loop mutations and observed significant differences of amber codon suppression level among the variants (36–39). Recently, we also reported that anticodon stem mutations of initiator tRNA also affect the efficiency of introducing noncanonical initiator building blocks, such as *N*-acetylproline and *N*-acetyl-β-homophenylglycine, at the peptide N-terminus (40). Therefore, here we hypothesize that the efficiency of β-amino acid elongation can also be finetuned by introducing mutations into the anticodon arm. Such an optimization of tRNA^Pro1E2^ would enable more efficient β-amino acid incorporation, leading to simultaneous introduction of multiple kinds of β-amino acids.

## Materials and methods

### Preparation of tRNAs and flexizymes

tRNAs (tRNA^Pro1E2^ variants and tRNA^iniP^) and flexizymes (dFx and eFx) used in this study were synthesized by *in vitro* transcription. The DNA templates for transcription were prepared by extension and PCR using the forward and reverse primers shown in Supplementary Table S1. In vitro transcription was carried out at 37°C for overnight in the following solution: 40 mM Tris–HCl (pH 8.0), 3.75 mM nucleoside triphosphates (NTPs), 5 mM guanosine monophosphate (GMP), 22.5 mM MgCl_2_, 0.01% Triton X-100, 1 mM spermidine, 1 mM dithiothreitol, 0.04 U/μl RNasin RNase inhibitor (Promega) and 0.12 μM T7 RNA polymerase. For transcription of flexizymes, the NTP concentration was increased to 5 mM and instead GMP was omitted. Then, the reaction mixture was treated with RQ1 DNase (Promega) for 30 min at 37°C. The resulting tRNA and flexizymes were purified by 8% or 12% denaturing polyacrylamide gel electrophoresis.

### Aminoacylation of tRNAs

3,5-Dinitrobenzylester (DBE) of βPhg, βMet, βGln, (1*S*,2*S*)-2-ACPC, (1*R*,2*R*)-2-ACPC, ^NO^Ala and ^NO^Gly and cyanomethyl ester (CME) of ^ClAc^d-Tyr were synthesized by the previously reported methods (26,35,41,42). We used dFx for aminoacylation of DBE-activated amino acids on tRNA^Pro1E2^ variants and eFx for acylation of ^ClAc^d-Tyr-CME on tRNA^iniP^_CAU_. Aminoacylation reactions of βPhg, βMet, βGln, (1*S*,2*S*)-2-ACPC, (1*R*,2*R*)-2-ACPC, ^NO^Ala and ^NO^Gly were performed for 24 h and that of ^ClAc^d-Tyr performed for 2 h at 4°C in the following reaction mix: 50 mM Bicine–KOH (pH 9.0, 8.7, or 8.5) or HEPES–KOH (pH 7.5), 600 mM MgCl_2_, 20% dimethyl sulfoxide, 25 μM eFx or dFx, 25 μM tRNA, and 5 mM activated amino acid. Acylation of βPhg, βMet and βGln was performed at pH 9.0, (1*S*,2*S*)-2-ACPC and (1*R*,2*R*)-2-ACPC at pH 8.7, ^NO^Ala and ^NO^Gly at pH 8.5, and ^ClAc^d-Tyr at pH 7.5. The resulting aminoacyl-RNAs were recovered by ethanol precipitation and washed with 70% ethanol.

### Ribosomal incorporation of β-amino acids into model peptides

*In vitro* translation of model peptides was carried out at 37°C for 30 min using a custom-made *Escherichia coli* reconstituted translation system, referred to as the flexible *in vitro* translation (FIT) system, in the following reaction mixture (25): 50 mM HEPES–KOH (pH 7.6), 100 mM potassium acetate, 12.8 mM magnesium acetate, 2 mM ATP, 2 mM GTP, 1 mM CTP, 1 mM UTP, 20 mM creatine phosphate, 0.1 mM 10-formyl-5,6,7,8-tetrahydrofolic acid, 2 mM spermidine, 1 mM dithiothreitol, 1.5 mg/mL *E. coli* total tRNA, 1.2 μM *E. coli* ribosome, 0.6 μM methionyl-tRNA formyltransferase, 2.7 μM IF1, 0.4 μM IF2, 1.5 μM IF3, 0.26 μM EF-G, 10 μM EF-Tu/EF-Ts, 5 μM EF-P, 0.25 μM RF2, 0.17 μM RF3, 0.5 μM RRF, 4 μg/ml creatine kinase, 3 μg/ml myokinase, 0.1 μM inorganic pyrophosphatase, 0.1 μM nucleotide diphosphate kinase, 0.5 μM DNA template, 0.1 μM T7 RNA polymerase, 0.73 μM AlaRS, 0.03 μM ArgRS, 0.38 μM AsnRS, 0.13 μM AspRS, 0.02 μM CysRS, 0.06 μM GlnRS, 0.23 μM GluRS, 0.09 μM GlyRS, 0.02 μM HisRS, 0.4 μM IleRS, 0.04 μM LeuRS, 0.11 μM LysRS, 0.03 μM MetRS, 0.68 μM PheRS, 0.16 μM ProRS, 0.04 μM SerRS, 0.09 μM ThrRS, 0.03 μM TrpRS, 0.02 μM TyrRS, 0.02 μM ValRS, 0.5 mM Asp, 0.5 mM Gly, 0.5 mM Lys, 0.5 mM Met, 0.5 mM Tyr and 20 μM each pre-charged β-aminoacyl-tRNAs. For translation of P3—10-βPhg, concentration of βPhg-tRNA was increased to 50 μM. For translation of P12, 50 μM of ^ClAc^d-Tyr-tRNA^iniP^_CAU_ and 0.5 mM Cys were also added to the above solution and 10-formyl-5,6,7,8-tetrahydrofolic acid and Met were removed. In addition, the concentrations of β-aminoacyl-tRNAs and EF-Tu/EF-Ts were increased to 40 and 100 μM, respectively. For radiolabelling of peptides, 0.05 mM of [^14^C]-Asp was used in place of cold Asp. The DNA templates for translation were prepared by extension and PCR using the corresponding forward and reverse primers (Supplementary Table S1). Since the FIT system contains T7 RNA polymerase, the DNA templates were transcribed into mRNA and then translated into peptides in the system.

### Tricine SDS-PAGE of translated peptides and their quantification by autoradiography

Translation was conducted in the presence of 0.05 mM [^14^C]-Asp in place of cold Asp, quenched by adding the same volume of stop solution [0.9 M Tris–HCl (pH 8.45), 8% SDS, 30% glycerol, and 0.001% xylene cyanol], and incubated at 95°C for 3 min. 4 μl of the sample was subjected to 15% tricine SDS-PAGE and analyzed by autoradiography using a Typhoon FLA 7000 (Cytiva). The relative translation efficiencies of peptides were estimated by the autoradiographic intensity of the peptide band in comparison with that of the control experiment, whose relative level was defined as 1.

### Identification of translated peptides by MALDI-TOF mass spectrometry

5 μl of translation reaction mix was desalted using SPE C-tip (Nikkyo Technos) and co-crystallized with α-cyano-4-hydroxycinnamic acid on a sample plate. The sample was analyzed by UltrafleXtreme (Bruker Daltonics) in a reflector/positive mode. Peptide calibration standard II (Bruker Daltonics) was used for external mass calibration.

## Results

### Evaluation of anticodon stem mutations for consecutive incorporation of β-amino acids at CCG codon

**Figure 2.**
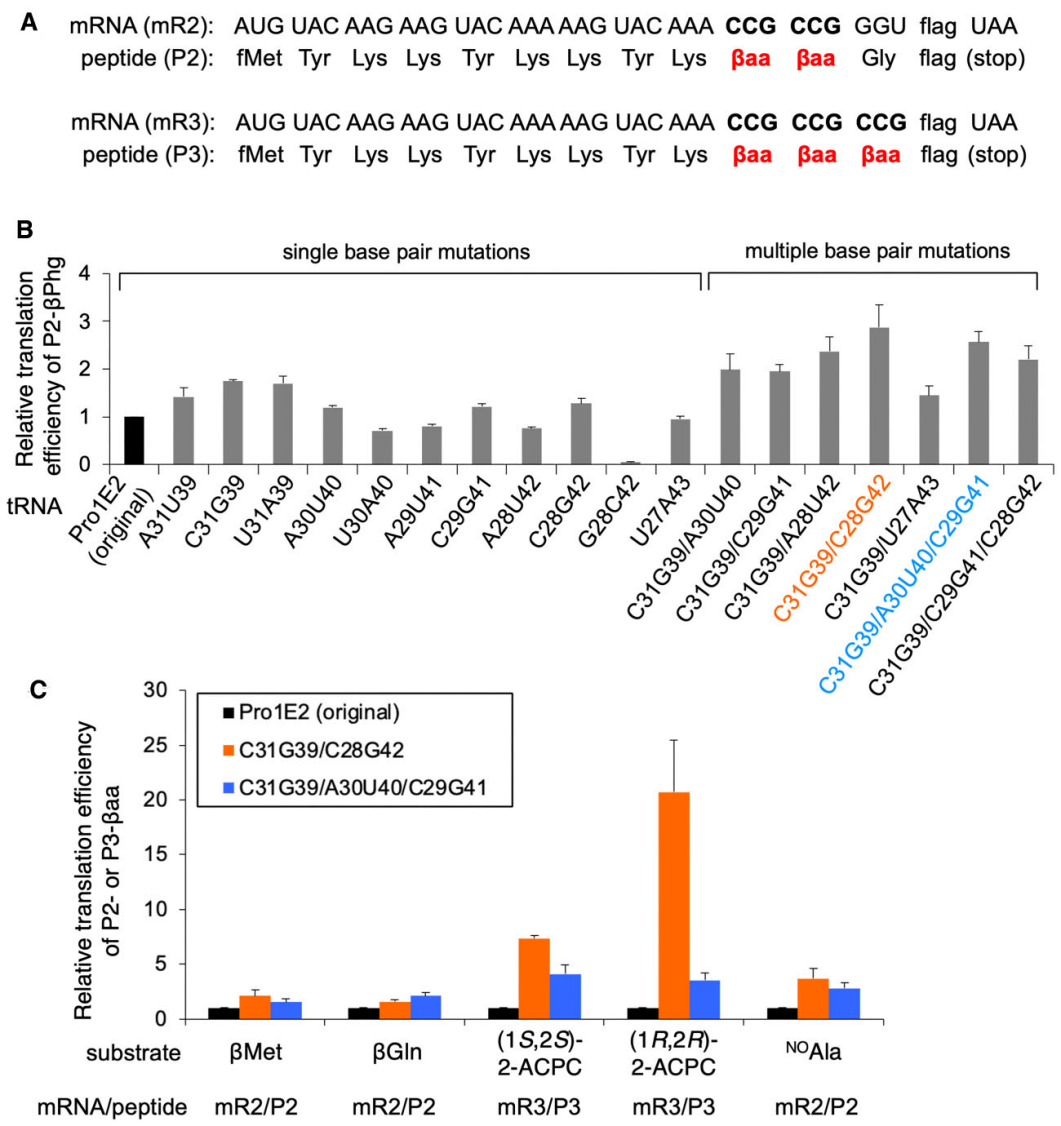
Ribosomal incorporation of β-amino acids into model peptides using tRNA^Pro1E2^_CGG_ and its anticodon stem variants. (**A**) mRNAs, mR2 and mR3, and the corresponding peptide sequences, P2 and P3, used for β-amino acid (βaa) incorporation. The amino acid sequence of ‘flag’ is Asp-Tyr-Lys-Asp-Asp-Asp-Asp-Lys. (**B**) Relative translation efficiency of peptide P2-βPhg. βPhg was assigned at the CCG codons of mR2 using pre-charged βPhg-tRNA, where the original tRNA^Pro1E2^_CGG_ (Figure 1A) and its anticodon stem mutants were used. Mutations at the anticodon stem are indicated at the bottom. The efficiency of the original tRNA^Pro1E2^_CGG_ was defined as 1. The variants that showed the highest and the second highest P2-βPhg levels are indicated by orange and blue, respectively. Error bars, s.d. (*n* = 3). See also Supplementary Figure S2A for the raw data of tricine SDS-PAGE analyses of peptides. (**C**) Relative translation efficiency of peptide P2 and P3 bearing various β-amino acids. β-amino acids were assigned at the CCG codons of mR2 or mR3 using pre-charged β-aminoacyl-tRNAs. The original tRNA^Pro1E2^_CGG_ (black), C31G39/C28G42 (orange), and C31G39/A30U40/C29G41 (blue) were tested for their incorporations. Values for the original tRNA^Pro1E2^_CGG_ were defined as 1. Error bars, s.d. (*n* = 3). See also Supplementary Figure S2B for the raw data of tricine SDS-PAGE analyses of peptides.

For fine-tuning tRNA^Pro1E2^, we first focused on mutations at the anticodon stem. Two consecutive β-homophenylglycine (βPhg) residues were incorporated into a model peptide P2 at CCG codons of a template mRNA, mR2 (Figures 1B and 2A, The P2 peptide containing βPhg is referred to as P2-βPhg). The original tRNA^Pro1E2^ bearing CGG anticodon (Figure 1A, tRNA^Pro1E2^_CGG_) and 11 anticodon stem mutants were evaluated for βPhg incorporation. The original tRNA^Pro1E2^_CGG_ has 5 bp in the anticodon stem (Figure 1A, Supplementary Figure S1, C27G43, U28A42, U29A41, C30G40 and G31C39) and the compensatory mutations were introduced at one of the five base pairs, where the combinations of nucleotides were arbitrarily chosen (Figure 2B, Supplementary Figure S1, A31U39, C31G39, U31A39, A30U40, U30A40, A29U41, C29G41, A28U42, C28G42, G28C42 and U27A43). βPhg was precharged onto the tRNA^Pro1E2^_CGG_ variants by means of flexizyme (22) and then subjected to translation. For translation of peptides, we used a custom-made FIT system that contains a minimal set of amino acids required for translation of P2, i.e. Asp, Gly, Lys, Met and Tyr. The peptides were expressed in the presence of [^14^C]-Asp, subjected to 15% tricine SDS-PAGE, and quantified by autoradiography (Supplementary Figure S2A). The identities of peptides expressed in the presence of cold Asp instead of [^14^C]-Asp were also confirmed by MALDI-TOF mass spectrometry (MS) (Supplementary Figure S3A). The relative translation efficiencies of P2-βPhg using the anticodon stem mutants were estimated, where the efficiency of the original tRNA^Pro1E2^_CGG_ was defined as 1 (Figure 2B). Among the 11 single base pair mutants, A31U39, C31G39, U31A39, A30U40, C29G41 and C28G42 showed higher expression levels of P2-βPhg compared to the use of the original tRNA^Pro1E2^_CGG_ (Figure 2B, 1.4-, 1.7-, 1.7-, 1.2-, 1.2- and 1.3-fold, respectively). All mutations at the N31N39 base pair resulted in higher level of P2-βPhg, indicating that the original G31C39 pair is not optimal for βPhg incorporation at CCG codon. C31G39 showed the highest translation efficiency of P2-βPhg, a 1.7-fold enhancement compared to the original tRNA^Pro1E2^_CGG_.

To further improve the translation efficiency, we next tried combinations of mutations that resulted in high relative translation efficiencies (Figure 2B, combinations of C31G39, A30U40, C29G41 and C28G42). Here we evaluated five double base pair mutants (C31G39/A30U40, C31G39/C29G41, C31G39/A28U42, C31G39/C28G42 and C31G39/U27A43) and two triple base pair mutants (C31G39/A30U40/C29G41 and C31G39/C29G41/C28G42). As a result, C31G39/C28G42 and C31G39/A30U40/C29G41 showed the highest and second highest P2-βPhg levels (Figure 2B, Supplementary Figure S2A, S3A, 2.9- and 2.6-fold enhancement compared to the original tRNA^Pro1E2^_CGG_, respectively). The relative P2-βPhg levels of the 19 tRNA^Pro1E2^_CGG_ variants tested in this study ranged from 0.05 to 2.9, and their average and variance values were 1.5 and 0.5, respectively, showing the impact of anticodon stem mutation on the efficiency of βPhg incorporation.

To evaluate the applicability of these anticodon stem mutants to introduce other β-amino acids and β-amino acid analogs, two β^3^-amino acids (βMet and βGln), two cyclic β^2,3^-amino acids [(1*S*,2*S*)-2-ACPC and (1*R*,2*R*)-2-ACPC] and one α-aminoxy acid (^NO^Ala) were precharged on C31G39/C28G42 and C31G39/A30U40/C29G41 as well as the original tRNA^Pro1E2^_CGG_ as a control and introduced into P2 or P3 at CCG codons (Figures 1B and 2C, Supplementary Figure S2B). Consequently, both C31G39/C28G42 and C31G39/A30U40/C29G41 showed significantly higher translation efficiencies of peptides compared to the use of the original tRNA^Pro1E2^_CGG_ for all substrates (Figure 2C, 1.5−20-fold enhancement), indicating that these mutant tRNAs can generally enhance incorporation of β-amino acids/analogs when introduced at CCG codon. The identities of these peptides were also confirmed by MALDI-TOF MS, showing that all these β-amino acids/analogs could be correctly introduced without misincorporation (Supplementary Figure S3B).

**Figure 3.**
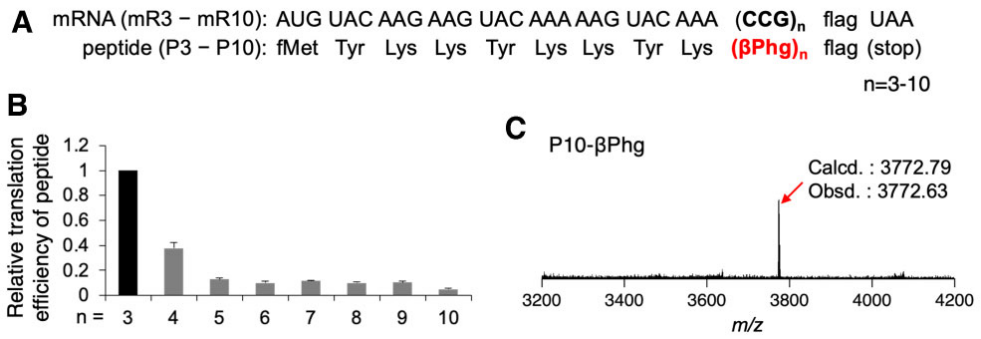
Consecutive incorporation of βPhg into model peptides. (**A**) mRNAs, mR3−mR10, and the corresponding peptide sequences, P3−P10, used for βPhg incorporation. (**B**) Relative translation efficiency of peptides P3−P10 bearing βPhg. The translation efficiency of P3-βPhg was defined as 1. Error bars, s.d. (*n* = 3). See also Supplementary Figure S2C for the raw data of tricine SDS-PAGE analyses of peptides. (**C**) MALDI-TOF MS of peptide P10-βPhg. ‘Calcd.’ and ‘Obsd.’ indicate calculated and observed *m/z* values, respectively. See also Supplementary Figure S3B for P3−P9.

By using C31G39/C28G42, we next tried three or more consecutive incorporations of βPhg into model peptides P3−P10 at CCG codon (Figure 3A). Although their translation efficiencies gradually decreased for more consecutive βPhg incorporations, up to ten consecutive incorporations of βPhg into P10 was confirmed (Figure 3B, C, Supplementary Figure S2C, the translation level of P3 was defined as 1, and the relative levels of P4 − P10 were calculated). The identities of the peptides were also confirmed by MALDI-TOF MS (Figure 3C, Supplementary Figure S3C). This is the first demonstration of ten consecutive incorporation of a β^3^-amino acid to the best of our knowledge.

### Anticodon stem fine-tuning for βPhg incorporation at other codons

**Figure 4.**
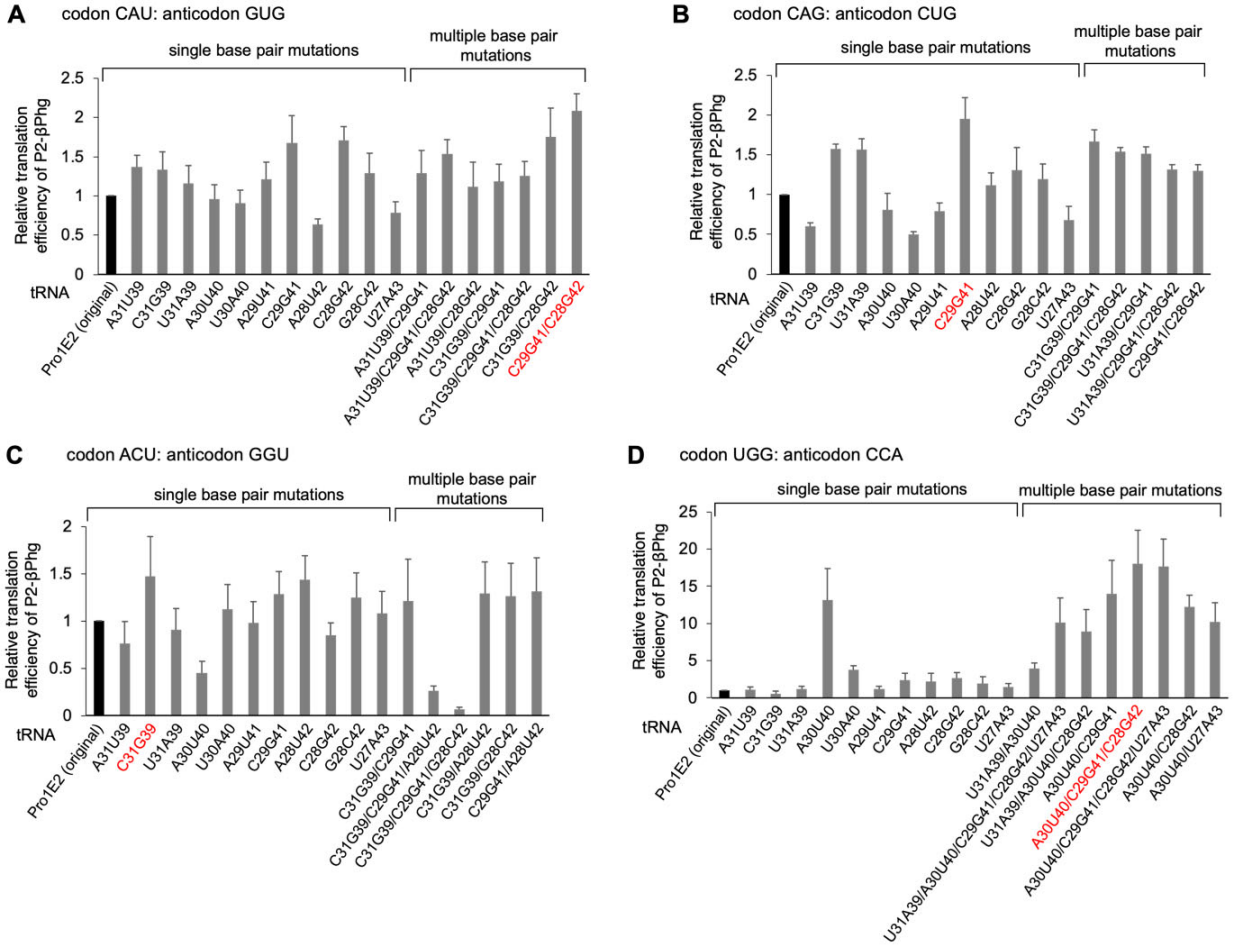
Ribosomal incorporation of βPhg into P2 using tRNA^Pro1E2^ and its anticodon stem variants. Introduction of βPhg at CAU (**A**), CAG (**B**), ACU (**C**) and UGG (**D**) codons of mR2 using pre-charged βPhg-tRNA^Pro1E2^ bearing GUG, CUG, GGU and CCA anticodons, respectively. The original tRNA^Pro1E2^ and its anticodon stem mutants were used. Mutations at the anticodon stem are indicated at the bottom. Peptides were analyzed by tricine SDS-PAGE and their translation efficiencies were quantified by autoradiography. Error bars, s.d. (*n* = 3). The variants that showed the highest P2-βPhg levels are indicated by red.

For simultaneous introduction of multiple kinds of β-amino acids/analogs into a single peptide, those amino acids must be assigned at different codons in a reprogrammed genetic code. Therefore, we needed to prepare multiple tRNA^Pro1E2^ variants bearing different anticodons. Here, we aimed at optimizing anticodon stem sequences for P2-βPhg incorporation at four codons in addition to CCG (Figure 4, CAU, CAG, ACU and UGG). These codons were decoded by anticodons of GUG, CUG, GGU and CCA, respectively. The anticodon loop sequence was comprised of 5′-UU-XXX-GA-3′, where XXX corresponds to the anticodon triplet. For the anticodon stem optimization, we took the same strategy as described above for CCG codon, i.e. evaluation of single base pair mutations followed by combinations of the effective single base pair mutations (Figure 4). For introduction of βPhg at CAU codon by GUG anticodon, A31U39, C31G39, C29G41 and C28G42 resulted in higher translation efficiencies of P2-βPhg compared to the original tRNA^Pro1E2^_GUG_ (Figure 4A, 1.4-, 1.3-, 1.7- and 1.7-fold increase compared to the original for A31U39, C31G39, C29G41 and C28G42, respectively). Then, combinations of these mutations were also evaluated (Figure 4A, A31U39/C29G41, A31U39/C29G41/C28G42, A31U39/C28G42, C31G39/C29G41, C31G39/C29G41/C28G42, C31G39/C28G42 and C29G41/C28G42). Consequently, C29G41/C28G42 showed the highest P2-βPhg level among all tRNA^Pro1E2^_GUG_ variants (Figure 4A, 2.1-fold increase). Likewise, for βPhg incorporation at CAG codon, C31G39, U31A39, C29G41 and C28G42 resulted in higher translation efficiencies of P2-βPhg compared to the original tRNA^Pro1E2^_CUG_ (Figure 4B, 1.6-, 1.6-, 2.0- and 1.3-fold, respectively). Combinations of these mutations were also tested, but none of the multiple base pair mutants showed higher P2-βPhg levels than the best single base pair mutant, C29G41 (2.0-fold). For ACU codon, C31G39, C29G41, A28U42 and G28C42 showed higher P2-βPhg levels than the original (Figure 4C, 1.5-, 1.3-, 1.4- and 1.2-fold increase, respectively). Although multiple base pair mutations were also tested, none of them showed higher P2-βPhg level than the best single base pair mutant, C31G39 (1.5-fold). Regarding UGG codon, A30U40 mutation showed a 13-fold higher P2-βPhg level compared to the original tRNA^Pro1E2^_CCA_. Combinations of some single base pair mutations (U31A39, A30U40, C29G41, C28G42 and U27A43) were also evaluated, where A30U40/C29G41/C28G42 resulted in the highest P2-βPhg level (Figure 4D, 18-fold). Based on these results, we chose C29G41/C28G42, C29G41, C31G39 and A30U40/C29G41/C28G42 for β-amino acid incorporation at CAU, CAG, ACU and UGG codons, respectively.

In summary, mutations of C31G39/C28G42, C29G41/C28G42, C29G41, C31G39 and A30U40/C29G41/C28G42 resulted in 2.9-, 2.1-, 2.0-, 1.5- and 18-fold enhancement of βPhg incorporation into P2 at CCG, CAU, CAG, ACU and UGG codons, respectively, compared to the use of the original tRNA^Pro1E2^. The ranges of P2-βPhg levels using the anticodon stem variants tested in this study were 0.05−2.9-fold, 0.6−2.1-fold, 0.5−2.0-fold, 0.07−1.5-fold and 0.5−18-fold for CCG, CAU, CAG, ACU and UGG codons, respectively. These results show that the optimal anticodon stem sequences differ depending on the sequence of codon/anticodon used for β-amino acid incorporation, indicating the importance of optimization of anticodon stem structure for efficient β-amino acid incorporation.

### Anticodon loop fine-tuning for βPhg incorporation

**Figure 5.**
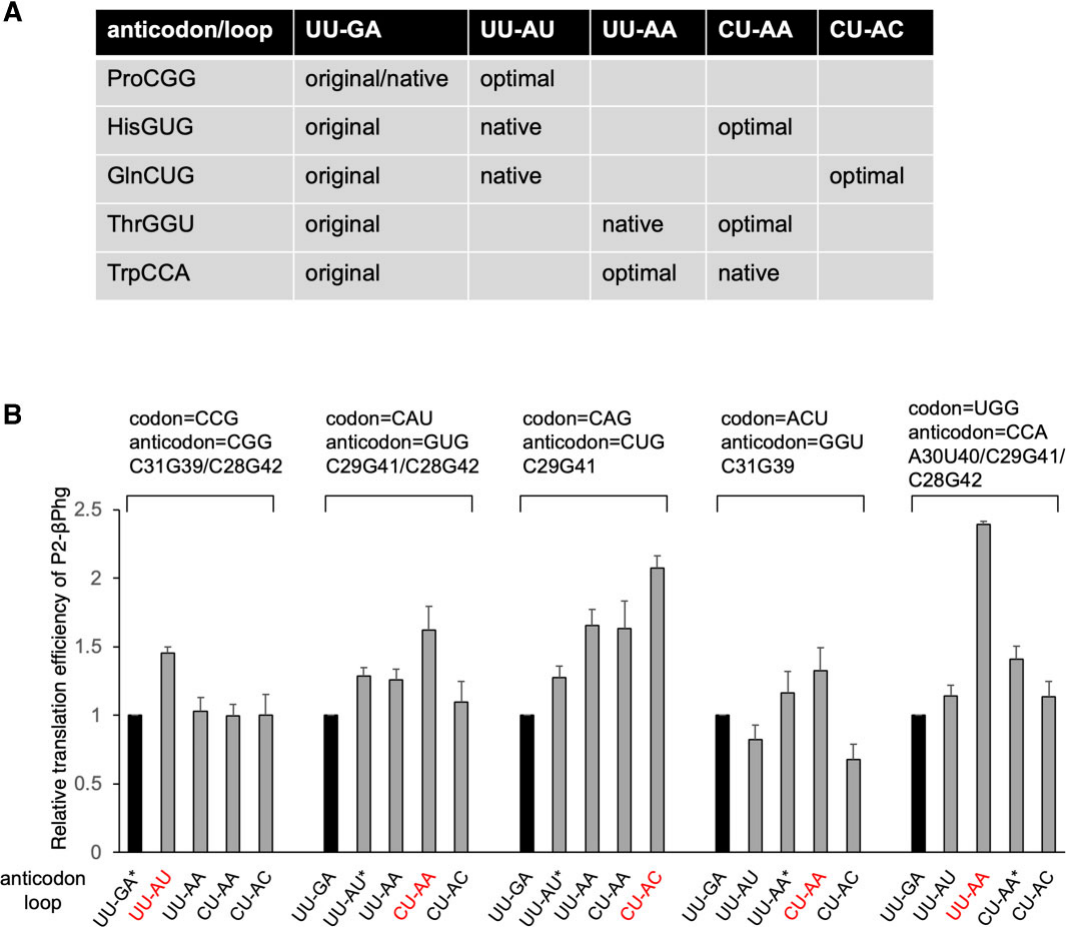
Ribosomal incorporation of βPhg into P2 using anticodon loop variants. (**A**) Combinations of tRNA anticodons and anticodon loop sequences tested in this study. The anticodon loop of the original tRNA^Pro1E2^ is UU-GA, which is indicated by ‘original’. The anticodon loop sequences of native *E. coli* tRNA^Pro1^_CGG_, tRNA^His^_GUG_, tRNA^Gln2^_CUG_, tRNA^Thr3^_GGU_ and tRNA^Trp^_CCA_ are indicated by ‘native’. The sequences that showed the highest translation efficiencies are indicated by ‘optimal’. (**B**) Relative translation efficiency of P2-βPhg using the anticodon-loop variants. The efficiencies of the original UU-GA were defined as 1. Error bars, s.d. (*n* = 3). The anticodon-loop sequences of the native *E. coli* tRNAs are indicated by asterisks. The mutations that showed the highest P2-βPhg levels are indicated by red.

Although we focused on anticodon stem sequences in the series of experiments described above, it is known that the anticodon loop sequence is also involved in regulating translation efficiency (36–39). Therefore, we next introduced anticodon loop mutations to the tRNA^Pro1E2^ variants bearing the optimal anticodon stem sequences to further enhance translation efficiency. The anticodon loop of the original tRNA^Pro1E2^ consists of 5′-UU-XXX-GA-3′, which is corresponding to the nucleotide positions of 32 to 38. In this mutation study, the nucleotides of positions 32, 33, 37 and 38 are changed to UU-AU, UU-AA, CU-AA and CU-AC. These sequences were chosen because the anticodon loop sequences of *E. coli* native tRNA^Pro1^_CCG_, tRNA^His^_QUG_, tRNA^Gln2^_CUG_, tRNA^Thr3^_GGU_ and tRNA^Trp^_CCA_ are UU-GA, UU-AU, UU-AU, UU-AA and CU-AA, respectively (Figure 5A). CU-AC was tested because this sequence is also found in other *E. coli* native tRNAs. These anticodon loop mutations were introduced into the anticodon stem variants of C31G39/C28G42, C29G41/C28G42, C29G41, C31G39 and A30U40/C29G41/C28G42 for two consecutive incorporations of βPhg into P2 at CCG, CAU, CAG, ACU and UGG codons, respectively (Figure 5B). Consequently, introduction of the UU-AU mutation into the C31G39/C28G42 variant resulted in 1.5-fold enhancement of βPhg incorporation at CCG codon compared to the original UU-GA, whereas other mutations, UU-AA, CU-AA and CU-AC, showed no significant enhancement. Thus, we decided to use the combination of the loop of UU-AU and the stem with C31G39/C28G42 mutations for incorporation of β-amino acids at CCG codon. Likewise, for the incorporation of βPhg at CAU, CAG, ACU and UGG codons, CU-AA, CU-AC, CU-AA and UU-AA mutations, respectively, resulted in the highest translation efficiencies of P2-βPhg (Figure 5B, 1.6-, 2.1-, 1.3- and 2.4-fold enhancement compared to the original anticodon loop, UU-GA). Therefore, these variants were chosen for β-amino acid incorporation at each codon.

### Introduction of five different β-amino acids/analogs into a model peptide at once

**Figure 6.**
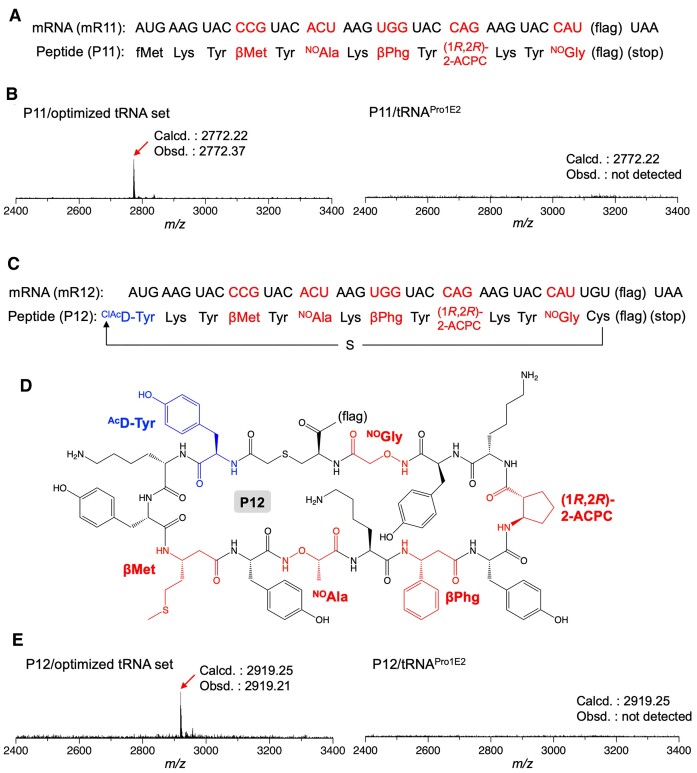
Introduction of five different β-amino acids at once. **(A, C)** mRNAs, mR11 and mR12 and the corresponding peptide sequences, P11 and P12. **(B, E)** MALDI-TOF MS of peptides P11 and P12. βPhg, βMet, (1*R*,2*R*)-2-ACPC, ^NO^Ala and ^NO^Gly were introduced at UGG, CCG, CAG, ACU and CAU codons, respectively, using the optimal anticodon-stem/loop variants (A30U40/C29G41/C28G42 + UU-AA, C31G39/C28G42 + UU-AU, C29G41 + CU-AC, C31G39 + CU-AA and C29G41/C28G42 + CU-AA, respectively) or the original tRNA^Pro1E2^ set. For translation of P12, ^ClAc^d-Tyr was introduced at the initiator AUG codon using tRNA^iniP^_CAU_. The thiol group of the downstream Cys attacks the N-terminal chloroacetyl group to form a thioether bond, yielding a macrocyclic structure. (**D**) Structure of P12.

To demonstrate the potency of the optimized tRNA^Pro1E2^ sets bearing the fine-tuned anticodon arms, five different β-amino acids/analogs were introduced into a model peptide, P11, at once (Figure 6A). βPhg, βMet, (1*R*,2*R*)-2-ACPC, ^NO^Ala and ^NO^Gly were precharged on the tRNA^Pro1E2^ variants bearing anticodons of CCA, CGG, CUG, GGU and GUG and anticodon stem/loop mutations of A30U40/C29G41/C28G42 + UU-AA, C31G39/C28G42 + UU-AU, C29G41 + CU-AC, C31G39 + CU-AA and C29G41/C28G42 + CU-AA, and introduced at UGG, CCG, CAG, ACU and CAU codons of mR11, respectively. Consequently, the expression of the desired peptide, P11, was confirmed by MALDI-TOF MS (Figure 6B left), whereas the use of the original tRNA^Pro1E2^ set for the five substrates yielded no desired product (Figure 6B right). We also demonstrated expression of a model macrocyclic peptide, P12, containing βPhg, βMet, (1*R*,2*R*)-2-ACPC, ^NO^Ala and ^NO^Gly (Figure 6C, D). These amino acids were introduced by using the same set of tRNA^Pro1E2^ variants as the expression of P11. For macrocyclization of the peptide, *N*-chloroacetyl-d-tyrosine (^ClAc^d-Tyr) was introduced at the initiator AUG codon in place of the canonical fMet using the engineered initiator tRNA referred to as tRNA^iniP^ (Supplementary Figure S4). EF-P recognizes the D-arm motif of tRNA^iniP^ to enhance incorporation of noncanonical initiator substrates such as d-amino acids and β-amino acids (40). The chloroacetyl group of ^ClAc^d-Tyr spontaneously reacted with the thiol group of the downstream Cys to form a thioether bond and eventually a macrocyclic scaffold. As a result, expression of the desired P12 in a macrocyclic form was confirmed by MALDI-TOF MS (Figure 6E). In contrast, the desired peptide was not observed when the original tRNA^Pro1E2^ set was used for incorporation of β-amino acids/analogs, showing the advantage of the anticodon arm-tuning strategy in incorporation of multiple β-amino acids/analogs.

## Discussion

In summary, we succeeded in fine-tuning anticodon arm structure of tRNA^Pro1E2^, thereby enhancing β-amino acid incorporation. The impact of anticodon arm mutation differed depending on the codons used for β-amino acid incorporation. Therefore, the anticodon arm sequence must be optimized individually for each codon.

In 1980s, Yarus et al. reported that substitution of anticodon loop nucleotides located at positions 32, 33, 37 and 38 affects *in vivo* translation efficiency and fidelity in *E. coli* (36–39). Later studies showed that these residues regulate the stability of codon-anticodon interaction *in vivo* and *in vitro* (43–48). Particularly, the 32–38 pair is a critical determinant of the binding affinity of aminoacyl-tRNA to the ribosomal A site; the affinity decreases by introducing a base pair at the 32–38. For instance, in the case of tRNA^Ala2^_GGC_, base pair formation at this position, such as A-U and C-G, is preferred for efficient decoding with high fidelity (46), whereas tRNA^Ala1^-based amber suppressor tRNAs bearing CUA anticodon exhibit higher suppression efficiencies with non-base paired 32–38 in the order of C-A > C-C > U-C > U-U (45). This is likely because the 32–38 pair contributes to fine-tuning the affinity to a uniform range. tRNA^Ala2^_GGC_ forms three G-C pairs between the codon and anticodon, resulting in high affinity, which is downregulated by introducing a base pair at the 32–38. On the other hand, the amber suppressor tRNA^Ala1^ bearing CUA anticodon forms only one G–C pair and thus the low affinity should be compensated for by the non-base-paired 32–38 for efficient translation. Oleiniczak *et al.* proposed that native tRNAs have evolved to have optimal 32–38 pairs for each anticodon so that the tRNAs have uniform affinities to the codon of mRNA (44,45).

However, these preceding studies focused on incorporation of canonical α-amino acids and thus the role of anticodon loop in β-amino acid incorporation had remained to be elucidated. As β-amino acids are extremely inefficient substrates for translation compared to the canonical α-amino acids due to their longer backbone, the anticodon loop sequences evolved for α-amino acid incorporation would not be necessarily optimal for β-amino acid incorporation. In fact, we revealed in this study that the native anticodon loop sequences were not optimal for β-amino acid incorporation (Figure 5). U–U, C–A, C–C, C–A and U–A were preferred for CGG, GUG, CUG, GGU and CCA anticodons, respectively, over the native loop sequences, U–A, U–U, U–U, U–A and C–A, respectively.

Although the role of anticodon stem in regulation of translation efficiency has not been fully elucidated to date, it is known that the anticodon stem is involved in modulating structural flexibility of tRNA. As tRNAs undergo conformational changes during translation on the ribosome, tRNA flexibility must be related to translation efficiency (49,50). We have speculated that, by modulating the flexibility of tRNA with anticodon stem mutations, β-amino acid could be placed at a preferable and reactive position at the peptidyl transferase center (PTC) of ribosome. The optimal location of β-amino acid for the reaction at the PTC may be different from that of the canonical α-amino acid due to the longer backbone of β-amino acid by one methylene group. Thus, we confirmed that the sequences of the anticodon stem/loop mutants obtained in this study were not shared by any *E. coli* native tRNAs that are used for α-amino acid incorporation. Another possible explanation would be stabilization of codon-anticodon interaction by the anticodon stem mutations, similar to the function of anticodon loop nucleotides as mentioned above. Further structural/biochemical studies are required to elucidate the mechanism how anticodon stem mutations regulate the β-amino acid incorporation efficiency.

By using the optimized anticodon arm variants, we have demonstrated ribosomal incorporation of five different types of β-amino acids/analogs at once as well as ten consecutive incorporation of βPhg for the first time, to the best of our knowledge. The advantage of ribosomal incorporation of β-amino acids is that we can easily prepare random peptide libraries bearing various β-amino acids using randomized mRNA templates. Such peptide libraries can be readily combined with mRNA display-based screening methodologies, such as the RaPID system, to efficiently screen bioactive peptides that bind to specific target molecules. By introducing β-amino acids into the libraries, we can expect enhanced binding affinity and specificity to target molecules, membrane permeability and proteolytic stability of the screened peptides.

## Supplementary Material

gkae219_Supplemental_Files

## Data Availability

The data underlying this article are available in the article and in its online supplementary material.
