# Supplementary material for: Fine-tuning the tRNA anticodon arm for multiple/consecutive incorporations of β-amino acids and analogs
Source: Nucleic Acids Res. 2024 Apr 4;52(11):6586–95. doi: 10.1093/nar/gkae219 (PMC11194099; doi:10.1093/nar/gkae219)
Supplement: gkae219_Supplemental_Files [file gkae219_supplemental_files.zip › 240224AStuning-Supplementary.pdf]

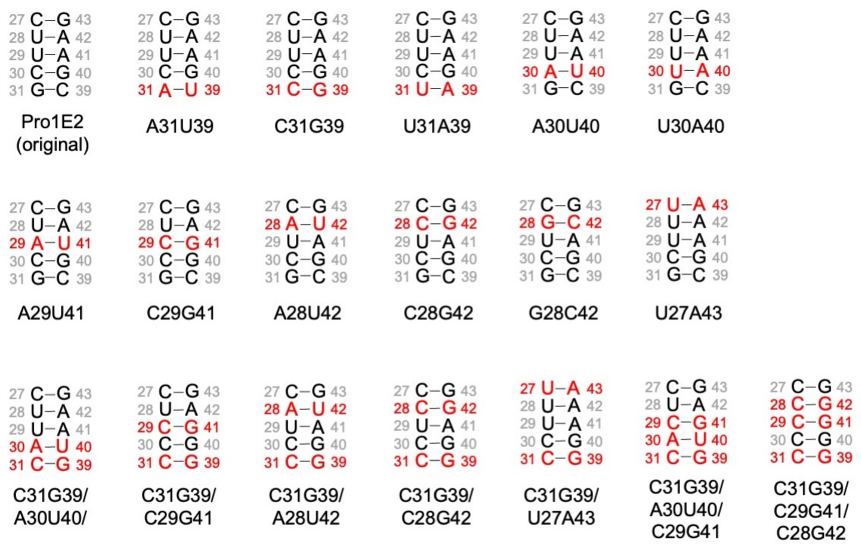

Supplementary Figure 1. Structures of the anticodon stem region of tRNA^{Pro1E2} variants.

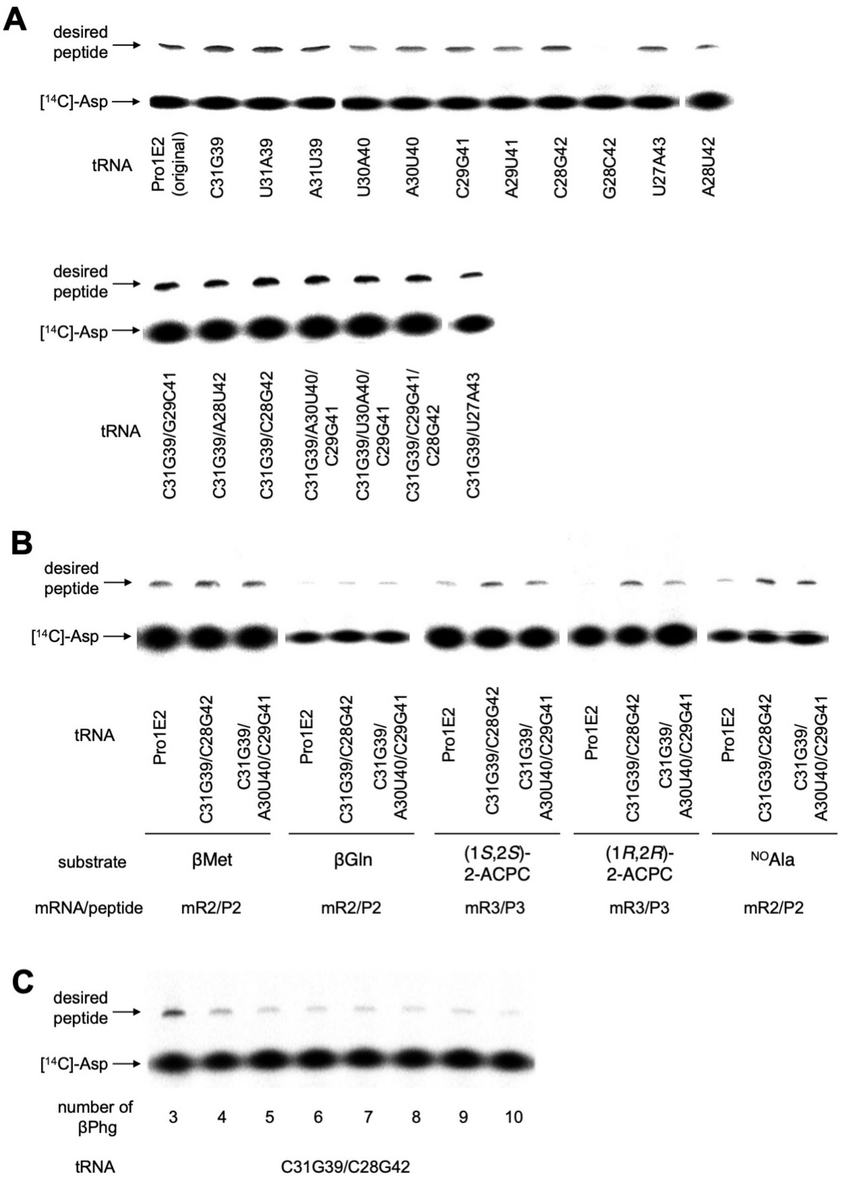

Supplementary Figure 2. Tricine SDS-PAGE analysis of translated peptides. (A) Introduction of βPhg into peptide P2 using $tRNA^{Pro1E2}_{CGG}$ and its variants. **(B)** Introduction of βMet, βGln, (1*S*,2*S*)-2-ACPC, (1*R*,2*R*)-2-ACPC, and ^{NO}Ala into P2 or P3. The original $tRNA^{Pro1E2}_{CGG}$, C31G39/C28G42, and C31G39/A30U40/C29G41 were tested for their incorporation. **(C)** Introduction of βPhg into peptide P3−P10 using C31G39/C28G42.

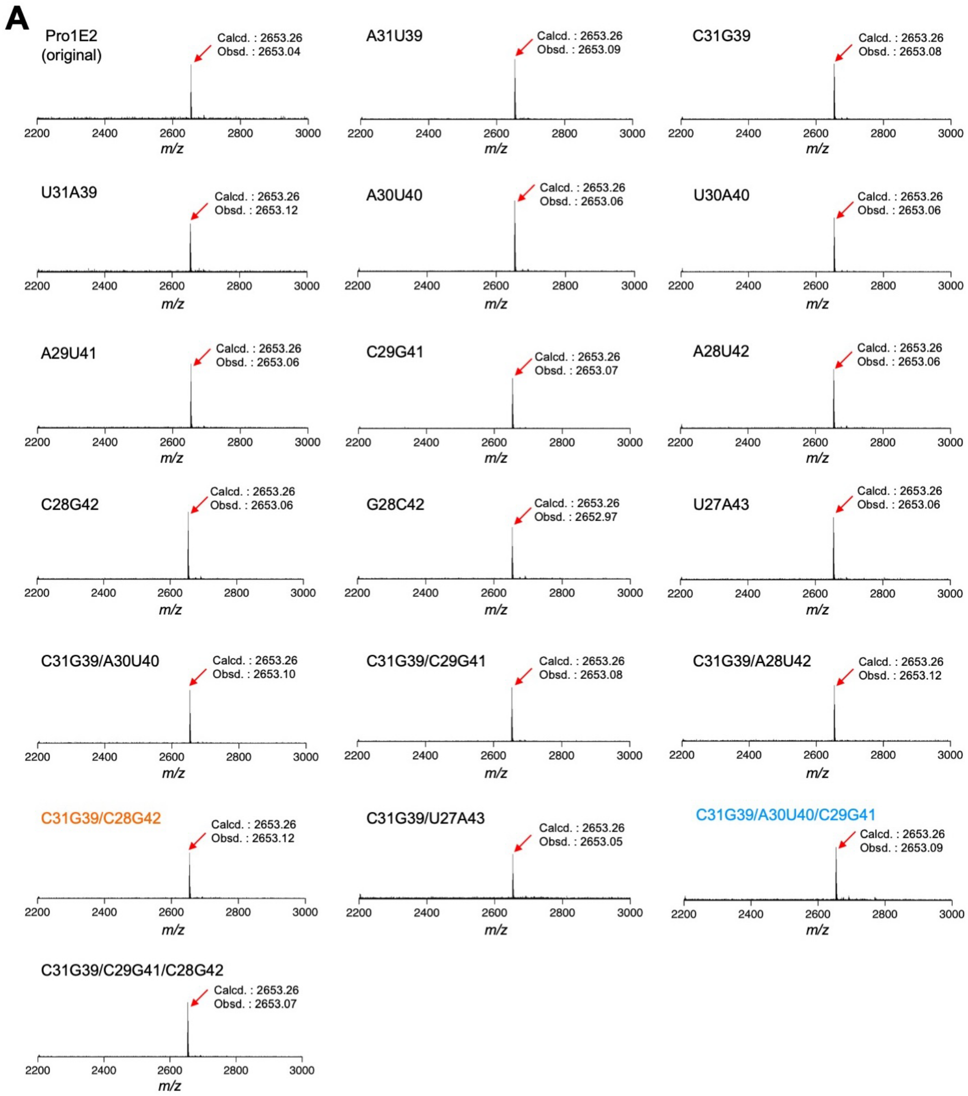

Supplementary Figure 3. MALDI-TOF MS of model peptides. (A) Introduction of βPhg into peptide P2 using $tRNA^{Pro1E2}_{CGG}$ and its variants. **(B)** Introduction of βMet, βGln, (1*S*,2*S*)-2-ACPC, (1*R*,2*R*)-2-ACPC, and ^{NO}Ala into P2 or P3. The original $tRNA^{Pro1E2}_{CGG}$, C31G39/C28G42, and C31G39/A30U40/C29G41 were tested for their incorporation. **(C)** Introduction of βPhg into peptide P3−P10 using C31G39/C28G42. 'Calcd.' and 'Obsd.' indicate calculated and observed *m/z* values, respectively.

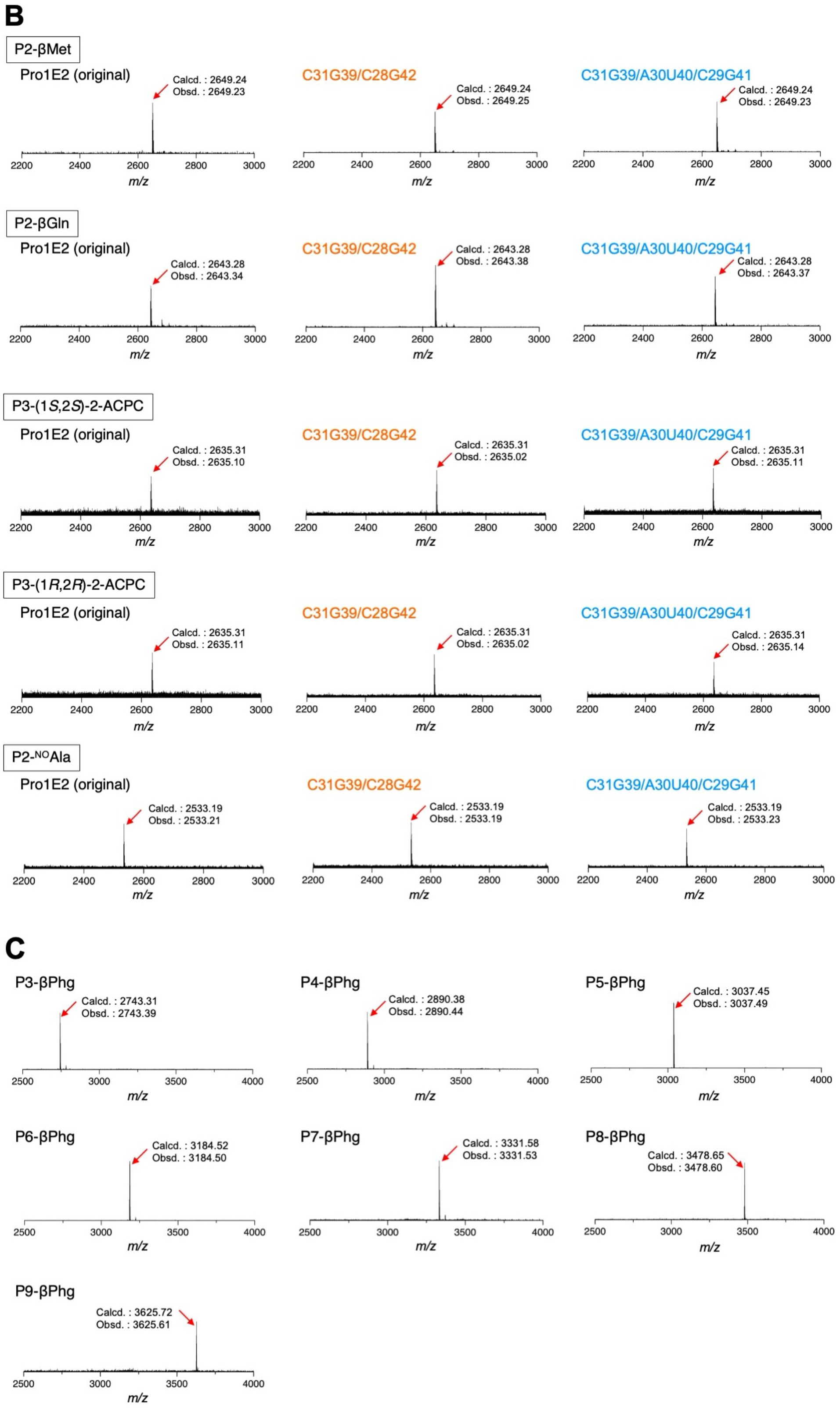

Supplementary Figure 3 continued.

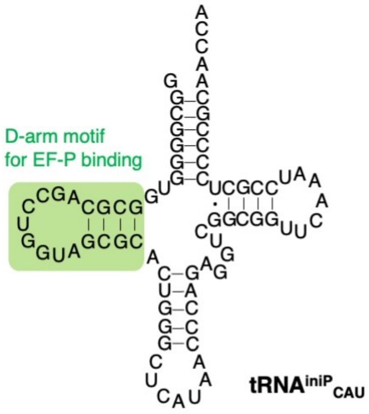

Supplementary Figure 4. Secondary structure of tRNA$^{iniP}_{CAU}$ that has a specific D-arm motif for EF-P binding. The body sequence of this tRNA, including anticodon stem, was previously optimized for incorporation of noncanonical initiator building blocks such as D-amino acids and β-amino acids (See reference 40 for details). This tRNA was used for incorporation of ^{ClAc}D-Tyr at the N-terminus of P12. See also Supplementary Table 1 for the sequence.
